# Assessing the properties of the prediction interval in random-effects meta-analysis

**DOI:** 10.1017/rsm.2025.10055

**Published:** 2026-01-09

**Authors:** Péter Mátrai, Tamás Kói, Zoltán Sipos, Nelli Farkas

**Affiliations:** 1 Institute of Bioanalysis, Medical School, University of Pécs, Pécs, Hungary; 2 Institute for Translational Medicine, Medical School, University of Pécs, Pécs, Hungary; 3 Department of Stochastics, Institute of Mathematics, Budapest University of Technology and Economics, Budapest, Hungary; 4 Centre for Translational Medicine, Semmelweis University, Budapest, Hungary

**Keywords:** coverage probability, heterogeneity, meta-analysis, prediction interval, random-effects, simulation

## Abstract

Random-effects meta-analysis is a widely applied methodology to synthesize research findings of studies related to a specific scientific question. Besides estimating the mean effect, an important aim of the meta-analysis is to summarize the heterogeneity, that is, the variation in the underlying effects caused by the differences in study circumstances. The prediction interval is frequently used for this purpose: a 95% prediction interval contains the true effect of a similar new study in 95% of the cases when it is constructed, or in other words, it covers 95% of the true effects distribution on average in repeated sampling. In this article, after providing a clear mathematical background, we present an extensive simulation investigating the performance of all frequentist prediction interval methods published to date. The work focuses on the distribution of the coverage probabilities and how these distributions change depending on the amount of heterogeneity and the number of involved studies. Although the single requirement that a prediction interval has to fulfill is to keep a nominal coverage probability on average, we demonstrate why the distribution of coverages should not be disregarded. We show that for meta-analyses with small number of studies, this distribution has an unideal, asymmetric shape. We argue that assessing only the mean coverage can easily lead to misunderstanding and misinterpretation. The length of the intervals and the robustness of the methods concerning the non-normality of the true effects are also investigated.

## Highlights

### What is already known?

- The routine report of a random-effects meta-analysis presents the estimated mean parameter and its confidence interval; however, these do not reveal the heterogeneity, that is, the differences in the underlying effects of individual studies.
- The prediction interval (PI) in random-effects meta-analysis is a useful tool to assess the heterogeneity; it intends to contain the true effect of a new similar study with a pre-specified probability.
- The Higgins–Thompson–Spiegelhalter PI method cannot keep the nominal mean coverage for cases when the number of studies is small, but the parametric bootstrap method can maintain appropriate mean coverage even if there are only three involved studies.

### What is new?

- If the number of involved studies is not large enough, the distribution of coverage probabilities is skewed. It means that with high probability, the coverage probability is close to 1; however, the mean coverage can still be close to the nominal level.
- The distribution of coverages reveals that even if the nominal mean coverage is maintained, the interpretation of a single published PI: “an interval that covers 95% of the true effects distribution” is not valid, because by definition, the PI covers this distribution only on average. In other words, if one considers a published PI with a nominal level of 0.95, it will not be true that approximately 95% of the upcoming individual studies will be within the published interval.

### Potential impact for RSM readers

- Even if the nominal mean coverage is approximately achieved, researchers using the PI should be cautious with its interpretation.
- Further research is needed regarding how new methods can be developed or how the currently existing PI methods can be altered to fulfill more strict conditions than the simple mean coverage criterion.

## 1 Introduction

Meta-analysis (MA) is a widely used scientific methodology that includes the process of systematically searching for and locating the quantitative evidence in a specific research question and integrating it using statistical methods. The most frequently applied meta-analysis model is the random-effects (RE) model, which assumes that the true effects in the distinct study populations are not equal, but they can be characterized by a common underlying distribution. Deviations between the true effects are generally referred to as heterogeneity, and it can be attributed to differences in study settings and populations. Almost all MAs focus on estimating a relative effect, for example, the effect of a specific intervention relative to a placebo; however, for simplicity, we will refer to such relative effects as “effect,” keeping in mind the relative nature of such estimates.

The conventional report of the frequentist random-effects model focuses on the point and interval estimation of the mean parameter of the true effects. The problem with this practice is that the mean parameter and its confidence interval (CI) give an estimate only for the mean of the true effects, and in the presence of heterogeneity, there might be study populations where the effect size is crucially different.^1^ ^–^ ^4^ Focusing only on the mean parameter hides these differences and can lead to overconfident, oversimplified, and potentially misleading conclusions.

In 2009, Higgins et al.^5^ proposed a prediction interval (PI) for the random-effects meta-analysis, which is a natural, straightforward way to summarize and report heterogeneity. In the past years, many authors have argued that the PI should be routinely part of an RE MA report,^2^ ^–^ ^4^ ^,^ ^6^ ^,^ ^7^ and indeed, the PI became a default or at least optional setting in many MA software. This interval has the advantage that it is on the same scale as the effect measure and contributes to a more complete summary of the meta-analysis. The 95% PI is an interval that covers 95% of the true effects distribution on average in repeated sampling, or phrased differently, the PI contains the true effect of a hypothetical, similar new study in 95% of the cases when it is constructed. We will point out that some commonly used PI interpretations are wrong: by definition, a 95% PI covers the true effects distribution only on average, and it is not true that all 95% PIs have a coverage probability of 95%. In this article, we go beyond the mean coverage probability by also investigating the distribution of coverages. We will show that investigating the distribution of the coverage probabilities reveals important aspects of the PI, which remain hidden if we assess only the mean coverage.

We found five simulation studies that aim to investigate the coverage performance of any PI estimator.^1^ ^,^ ^8^ ^–^ ^11^ These assess PI performance based on the probability that the random interval contains a newly generated true effect. Our study analyzes the PI from different aspects. We present a comprehensive simulation that we conducted to assess the performance of all frequentist PI methods that we could find in the literature. We investigate the Higgins–Thompson–Spiegelhalter (HTS) PI^5^ along with two newly proposed PI estimators, the one proposed by Wang and Lee,^12^ and a parametric bootstrap method proposed by Nagashima et al.^8^ We also investigate how sensitive these estimators are for the case when the random-effects distribution departs from normal.^5^ ^,^ ^13^ ^–^ ^15^

The structure of this article is the following: in Section 2, we review the RE model, the mathematical context of the meta-analytical PI. In Section 3, we give a formal definition of the PI, and we give a brief description of the investigated methods. In Section 4, we describe our simulation methods, and in Section 5, we present the results. In Section 6, we show an application of the various PI methods for a published MA, and we deal with the implications and concerns that arise during their interpretation. In the discussion in Section 7, we synthesize our findings and express our perception about the PI. In Section 8, we give a short conclusion.

## 2 Random-effects meta-analysis model

The random-effects meta-analysis model uses a probability distribution, so-called random effects, to model the differences in the circumstances of the published studies. There are often differences in the involved study populations (e.g., age, education, health status), they are often conducted in various countries with different cultures and health care systems, and the studied exposure and the follow-up times are also often meaningfully different.

In the mathematical model of the conventional two-step RE MA, it is assumed that the true (theoretical or population) effects of the involved studies are independent and identically distributed draws from an unknown distribution.^16^ In the conventional case, normality is assumed, and the main goal of the analysis is to estimate the expected value and the variance of this distribution.^17^ The published, observed outcomes are erroneous versions of the study-specific true effects. More exactly, it is assumed that a normally distributed random measurement error is added to the study-specific true effects. Let K denote the number of studies involved in the meta-analysis, and let 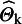 , 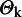 and 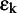 denote the observed outcome, the true effect, and the random error in the k-th study, respectively (k = 1, 2, …, K). Then, according to the basic model discussed above:
(1)

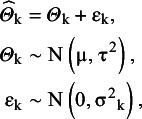

 where μ and 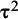 denote the expected value and the variance of the true-effects distribution, respectively, 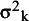 denotes the variance of the study-specific sampling error, and 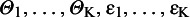 are independent.

If 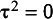 , the model above is called the common effect model (or fixed effect model). In this case, the assumption is made that each study estimates the same true effect and the observed effects differ only because of the within-study measurement error. It is very rare in social or medical sciences that the studies conducted with the same research question are so similar that the common effect model is plausible.^5^ Even if 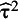 , the estimated between-study variance parameter is 0 for a collection of studies, it is reasonable to assume that we underestimate this variance, and some heterogeneity is still present in the given scientific field.

The conventional random-effects model specified under (1) makes two normality assumptions. The first one is that the observed effects are unbiased, normally distributed estimates of the unknown true effects. This assumption is well-founded for studies with a reasonable sample size, as the central limit theorem (CLT) or, for certain effect measures, the maximum likelihood theory guarantees at least asymptotic normality.^18^ The other normality assumption represented by the second line of (1) states that the true effects are also normally distributed with the same mean and variance parameter. This assumption is often criticized, as we can only argue with the CLT on this level of the model if we are willing to accept that the unexplained between-study variation is the sum of many independent factors.^13^ ^–^ ^15^

### 2.1 Parameter estimation in the random-effects model

The conventional aim of meta-analysis is to estimate μ and τ^2^. An unbiased estimate of μ can be given by a weighted mean: 
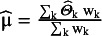
 . Weights are conventionally chosen as 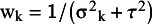 , because the variance of this estimator, 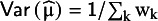 provides the uniformly minimum variance estimator for μ.^19^ Note that in the formula for weights, neither 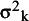 , nor 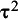 are known quantities. The standard approach treats 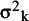 -s as fixed and known constants and replaces them with 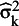 , the within-study variance estimates. Also, 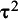 is replaced by its estimate, 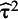 . Hence, in practice, weights are estimated as 
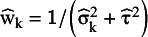
 and the estimate for 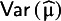 is calculated with these weights as 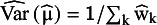 . This approach, like the within-study normality, is justifiable when the sample sizes are appropriately large.^6^ There are numerous methods in the literature for the estimation of the 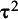 parameter, a good description of these can be found in Veroniki et al.^20^ We used two frequently applied 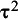 estimators to construct PI, the method of moments-based estimator suggested by DerSimonian and Laird (DL),^16^ and the restricted maximum likelihood (REML) estimator.^21^ Hartung and Knapp^22^ and Sidik and Jonkman^23^ independently proposed an alternative variance estimator for the μ parameter, denoted in the following by 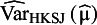 . This estimator takes into account that the 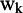 weights are not known but estimated quantities, and they use it with t distribution to construct a confidence interval for μ. 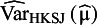 is usually larger than 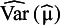 , but in some rare cases it can be the opposite way.

Heterogeneity is frequently assessed by the Q test introduced by Cochran,^24^ testing the null hypothesis of homogeneity ( 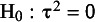 ), and by the I^2^ statistic or the magnitude of 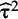 . The I^2^ statistic was proposed by Higgins and Thompson,^25^ and it can be interpreted as the proportion of total variance in the effect estimates that can be attributed to the between-study variance.

## 3 Prediction interval

When the between-study parameter estimate has a magnitude that is relevant in the specific research question, it means that μ, the average effect, and its confidence interval do not represent the quantity studied in an adequate way. In this case, there is a wide range of possible true effects that we can expect depending on unknown study conditions.^15^ These plausible true effects can be meaningfully different from the overall mean, but it is not reflected in the CI. One way to handle this situation is to make an attempt to explain this large heterogeneity by forming more homogenous subgroups or fitting a meta-regression model. Many times, such an attempt is not successful, and the researcher faces a substantial level of unexplained between-study variance at the end of the analysis. In this case, an important aim of the MA report is to present this uncertainty in the underlying true effects in an understandable and clear way. Higgins and his coauthors proposed a prediction interval in their 2009 paper,^5^ an interval that contains the true effect of a new study (conducted in similar conditions) in 95% of the cases when such an interval is constructed. It is possible to construct the PI with other than 95% probability, but we are approaching the PI mainly from a medical perspective, where 95% is the conventional case.

### 3.1 Formal definition of the prediction interval

We proceed to use the notation introduced in Section 2, stating that we have K studies that can be modelled by the data-generating process represented by (1). Let F denote the cumulative distribution function of the true effects distribution. The inputs of the meta-analysis are realizations of the random variables 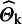 along with the corresponding within-study variances 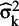 . Let D denote the input variables, that is, the collection of the random variables 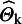 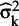 . Based on a realization of D, the applied PI method outputs a prediction interval; let L(D) and U(D) denote the lower and upper PI boundaries. Note that if a realization of D is given, the PI is a deterministic function of this realization. However, L(D) and U(D) are random variables, as the input data D can be considered as a 2K dimensional vector of random variables. The length of the interval can be defined as
(2)

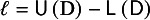

 and the covered probability as
(3)

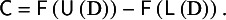



**Definition 1.** In random-effects meta-analysis, the random interval (L(D), U(D)) with E[C] = 1 – α is called a prediction interval with expected coverage probability 1 – α.

Let 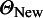 denote the true effect in a new study, that is, a random variable having the same distribution as 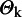 and which is independent of D. The tower rule implies that
(4)

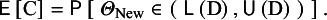



(For proof, see Appendix Proof 1 of the Supplementary Material). The above equality makes it clear that E[C] can be approximated by performing several times the following simulation procedure: simulating the input data D from model (1), calculating the interval (L(D), U(D)), simulating independently an additional true effect 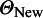 , then checking if 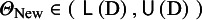 . If the number of simulations is large, the relative frequency of the cases when the calculated interval contains the newly generated true effect will be close to E[C]. All previous studies that investigated any PI method in the random-effects meta-analysis assessed the coverage performance only by approximating E[C] based on equation (4) using the above-described simulation approach.^1^ ^,^ ^8^ ^–^ ^11^

### 3.2 Methods to construct the prediction interval

#### 3.2.1 The Higgins–Thompson–Spiegelhalter (HTS) method

Higgins, Thompson, and Spiegelhalter were the first in their 2009 article to introduce the prediction interval in the context of the frequentist random-effects meta-analysis.^5^ They construct their PI estimator assuming 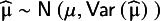 and 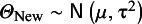 and the independence of 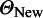 and 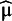 . These infer 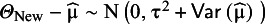 . They argue that as 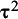 has to be estimated, and it impacts both parts of the variance of 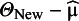 , the t-distribution with K − 2 degrees of freedom should be used to construct the PI. Assuming 
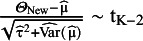
 , they propose the (1 – α) expected coverage level PI as
(5)

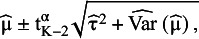

 where 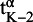 is the (1 − α/2) quantile of the t-distribution with K − 2 degrees of freedom.

Note that using the 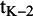 distribution has no convincing theoretical basis; Viechtbauer, for example, offers the standard normal distribution to construct the PI as the default method in his software.^26^ Partlett and Riley^9^ also used modified versions of (5) in their work.

#### 3.2.2 The parametric bootstrap method

Nagashima, Noma, and Furukawa published a bootstrap PI construction method,^8^ which is a parametric method because it utilizes heavily the initial assumption of the normality of random effects. They show that based on this assumption, 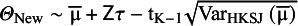 , where 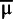 is an estimate for 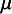 using the theoretical weights, assuming 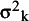 and 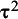 are known, Z is a random variable with standard normal distribution, t_K-1_ is a random variable with t distribution with K-1 degrees of freedom, and 
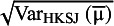
 is the theoretical standard error of 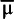 defined by Hartung and Knapp^22^ and Sidik and Jonkman.^23^ As a next step, they define a plug-in estimator for 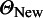 and give an approximate predictive distribution for 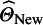 as
(6)

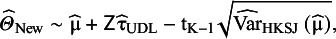

 where 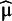 and 
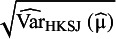
 are computed as 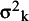 and 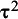 are estimated quantities, and 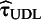 is the untruncated DerSimonian and Laird estimator for τ.^16^ As the formula for 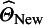 contains three random variables (Z, t_K-1_, and 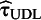 ), they generate B independent random realizations from these distributions and compute 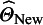 for each realization based on (6). Generating realizations from the distribution of 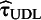 is difficult; it is based on the distribution of the Q statistics described by Biggerstaff and Jackson.^27^ For the boundaries of the (1 – α) expected coverage level PI, they simply take the α/2 and (1 − α/2) quantiles of the B realizations of 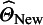 .

#### 3.2.3 The ensemble method

Wang and Lee^12^ used the background idea of Louis^28^ and constructed a PI estimator by modifying the observed effects so that the empirical distribution of 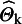 have a variance that equals 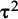 asymptotically. They define 
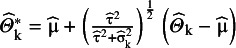
 and show that 
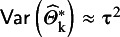
 for large K. As a next step, they simply use the α/2 and (1 − α/2) quantiles of 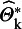 to create a PI with (1 – α) expected coverage level. Note that if 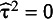 , this method yields a single number, 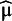 as PI, but the HTS type intervals in this case give an interval that is at least as wide as the CI.

### 3.3 PI estimators in common MA software

Since its introduction for the random-effects meta-analysis in the frequentist framework in 2009,^5^ the PI became an optional part in most of the commonly used MA software and program packages. The available and the default methods show a notable variety. We investigated the newest versions of the MA software that we think are commonly used and made a table showing the default and the other available PI methods (Table 1). The most common ones are the different HTS-type intervals. The parametric bootstrap method is only available in the meta R package.^29^ The ensemble method is not implemented in any of these software, but is available freely in a supplementary spreadsheet file published along with their article.^12^

**Table 1:** Default and other available prediction interval estimators in common meta-analysis software

Software	License type	Default PI method	Other available methods
R meta package v. 7.0–0	Freeware	HTS-REML (t_K–2_)	HTS-DL (t_K–2_) HTS-HKSJ (t_K–2_) Parametric bootstrap
R metafor package v. 4.8–0*	Freeware	HTS-REML (z)	HTS-DL (z) HTS-DL (t_K–1_) HTS-DL (t_K–2_) HTS-REML (t_K–2_) HTS-HKSJ (t_K–1_) HTS-HKSJ (t_K–2_)
Stata v. 18 meta command	Commercial	HTS-REML (t_K–2_)	HTS-DL (t_K–2_) HTS-HKSJ (t_K–2_)
Stata metan routine	Commercial	HTS-DL (t_K–2_)	HTS-REML (t_K–2_)
SPSS v. 29.0.2.0.	Commercial	HTS-REML (t_K–2_)	HTS-DL (t_K–2_) HTS-HKSJ (t_K–2_)
Comprehensive meta-analysis v. 4.	Commercial	HTS-DL (t_K–2_)	-
RevMan 9.3.0.	Freeware	HTS-HKSJ (t_K–1_)	HTS-REML (z) HTS-DL (z)

*Abbreviations:* HTS, Higgins–Thompson–Spiegelhalter method; REML, Restricted maximum likelihood estimation of 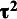 parameter; DL, DerSimonian and Laird estimation of 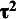 parameter; HKSJ, Hartung–Knapp–Sidik–Jonkman variance estimation for the μ parameter; t_K-2_, method calculated with t distribution with K-2 degrees of freedom; z, method calculated with standard normal distribution; “-,” denotes that the given option is not available.

* In metafor v. 4.8–0, the PI and CI are calculated based on the critical values from the standard normal distribution as the default setting. Otherwise, the PI is calculated based on the CI method selected, so that the PI is identical to the CI in the absence of observed heterogeneity. For more details, see the reference manual (https://cran.r-project.org/web/packages/metafor/metafor.pdf).

## 4 Simulation methods

Our meta-analysis input simulation approach is similar to the effect size simulation method of Bakbergenuly et al.^30^ We investigate a continuous variable and determine the mean difference (MD) as the effect measure between two groups, an experimental (E) and a control (C) group. We chose the mean difference as the effect measure in our simulation because it is a frequently used effect size in the medical field, but we argue that the conclusions are generalizable to other effect sizes (standardized mean difference, hazard ratio, odds ratio, risk ratio, etc.). Previous simulations show that the main factors influencing the PI performance are the number of involved studies and the amount of heterogeneity,^8^ ^,^ ^9^ regardless of the effect measure.

### 4.1 Generating study numbers and sample sizes

First, we fix K, the number of studies involved in the meta-analysis, as K in {3, 4, 5, 7, 10, 15, 20, 30, 100}; k represents the index of a given study in the meta-analysis, k = (1, 2, …,K). Then we determine the total sample size in a given study, N_k_, by one of two ways. The sample sizes are either equal in all included studies, in this case N_1_ = N_2_ = … = N_K_ in {30, 50, 100, 200, 500, 1000, 2000}, or if they are mixed, the vector (50, 100, 500) is repeated periodically to N_K_, namely N_1_ = 50, N_2_ = 100, N_3_ = 500, N_4_ = 50 … N_K_. For the sample sizes in the two groups, N_E, k_ and N_C, k_, we simply halve the total sample size: N_E, k_ = N_C, k_ = N_k_/2.

### 4.2 Simulation of standard errors

We fix the population variance (V) of the continuous variable in the two groups as V_E, k_ = V_C, k_ = 10. This does not lead to loss of generality, as we will control the standard error of the mean difference by controlling the sample size. As a next step, we calculate the theoretical variance of the mean difference, 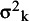 , and simulate its observed value in the sample, 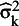 . For these, we first generate the sample variance, 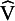 , in the two groups as a scaled random realization from a 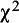 distribution with appropriate degrees of freedom: 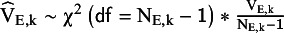 and 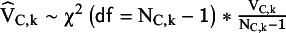 and compute the observed squared standard error as 
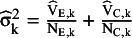
 and its theoretical version as 
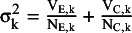
 .

### 4.3 Simulation of true effects and observed effects

To simulate the true effects, 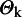 , and their observed value, 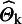 , we first fix τ^2^, the variance of the true effects as τ^2^ in {0.1, 0.2, 0.3, 0.5, 1, 2, 5}, and μ, the expected value of the true effects, as μ = 0. To simulate the true effects, we used the normal distribution and five other distributions to investigate how robust the methods are for the deviation from normality. We simulated 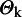 from slightly, moderately, and highly skewed skew-normal distributions with skewness coefficients of 0.5, 0.75, and 0.99, respectively. We also simulated true effects from a bimodal distribution mimicking the case where there is an underlying factor that divides the studies into two subgroups. We generated random numbers from a bimodal distribution as a mixture of two normals using the R package EnvStats v. 2.8.1.,^31^ and from the skew-normal distributions using the sn package v. 2.1.1.^32^ As an extremity, we also investigated the case where the true effects follow the uniform distribution. The density functions of these distributions are visualized in the Online Material file.^33^ As each investigated simulation factors are fully crossed, the number of the total investigated scenarios is as follows: (N) * (K) * (τ^2^) * (true effects distributions) = 8 * 9 * 7 * 6 = 3024.

### 4.4 Tested PI methods

We tested the parametric bootstrap method, the ensemble method, and six different versions of the HTS-type PI-s. The HTS-type methods differ in the τ^2^ estimator, the 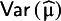 estimator, and the used distribution. Table 2 summarizes these versions with their notation used in the following. To all the HTS-type methods that are constructed with t distribution, we will refer simply as HTS (t).

**Table 2:** HTS-type prediction interval estimators tested in the simulation

Notation	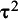 estimator	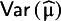 estimator	Distribution used
HTS-DL (t_K–2_)	DL	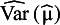	t (df = K–2)
HTS-REML (t_K–2_)	REML	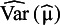	t (df = K–2)
HTS-HKSJ (t_K–2_)	REML	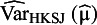	t (df = K–2)
HTS-DL (t_K–1_)	DL	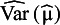	t (df = K–1)
HTS-HKSJ (t_K–1_)	REML	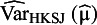	t (df = K–1)
HTS-DL (z)	DL	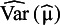	Standard normal

*Abbreviations:* HTS, Higgins–Thompson–Spiegelhalter method; REML, Restricted maximum likelihood estimation of 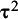 parameter; DL, DerSimonian and Laird estimation of 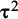 parameter; HKSJ, Hartung–Knapp–Sidik–Jonkman variance estimation for the μ parameter; t (df = K–2), method calculated with t distribution with K–2 degrees of freedom.

For each scenario, we simulated 5000 meta-analysis realizations and computed the PI with each investigated method, with the exception of the parametric bootstrap method, which we evaluated for 1000 realizations for each scenario. The parametric bootstrap method is a highly computationally intensive method requiring many bootstrap samples. This parameter can be set by the B parameter of the pima function in the pimeta R package. The default setting of the function is B = 25000, Nagashima et al. prepared their simulation with B = 5000 repetitions.^8^ We also used the B = 5000 setting in our simulation. We used the high-performance computing system of the Hungarian Governmental Agency for IT Development (KIFÜ) to evaluate the parametric bootstrap method and a simple personal computer for the other methods. The simulation was programmed in R v.4.1.3.^34^

### 4.5 Heterogeneity measures

We measured the heterogeneity of the scenarios in two ways. We calculated the ratio 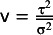 following Partlett and Riley,^9^ where 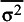 is the mean within-study variance. We also considered the mean of the observed I^2^ measures and used the DL method to estimate τ^2^ for the I^2^ computation. We chose our simulation parameters so that both v and I^2^ cover a wide range of values. Our investigated scenarios cover the range of 0.08–250 of v values and the 7%–99% range of the mean I^2^ values.

### 4.6 Investigated performance measures

We assessed the coverage performance of the PI methods based on the empirical distribution of the coverage probabilities defined by equation (3). We visualized and examined these distributions on histograms. We computed the mean of the coverage probabilities and the median coverage probability, approximating E[C] and Median[C], respectively. To gain more insight into the behavior of the investigated intervals, we also analyzed the length of the PI. To be able to compare the observed interval length (ℓ) to a reasonable ideal length, we defined T, the theoretical length, as the difference between the 0.975 and 0.025 quantiles of the true effects distribution. We assessed ℓ by comparing it to this theoretical length and investigated the mean of the observed length relative to the theoretical length, approximating 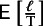 .

## 5 Simulation results

**Figure 1:**
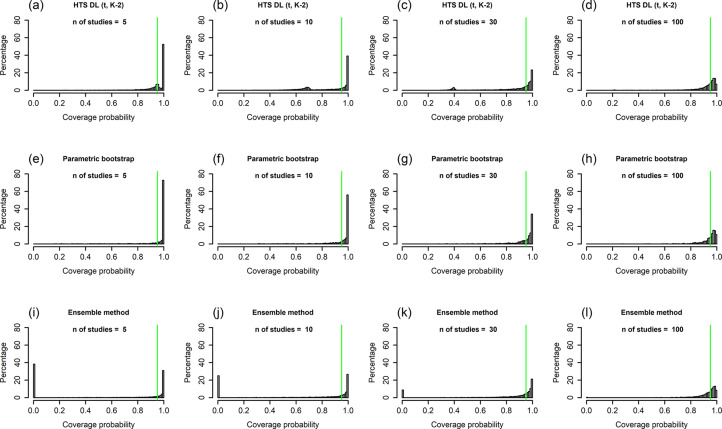
Histograms showing the coverage probability distribution of the HTS-DL (t_K-2_), the parametric bootstrap, and the ensemble prediction interval methods for a low heterogeneity simulation scenario (N = 100, τ^2^ = 0.2, I^2^ = 33%, v = 0.5). The vertical green lines on the histograms indicate 95% coverage probability. The number of involved studies is constant in each column, showing the distribution for 5, 10, 30, and 100 studies. Results for the HTS-DL (t_K-2_) method are represented in the first row of histograms with letters (a), (b), (c), and (d), the parametric bootstrap method is represented in the second row of histograms with letters (e), (f), (g), and (h), and the ensemble method is represented in the third row of histograms with letters (i), (j), (k), and (l). *Abbreviations*: HTS-DL (t_K-2_), Higgins–Thompson–Spiegelhalter method with the DerSimonian and Laird estimation of 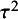 parameter and using the t distribution with K-2 degrees of freedom.

**Figure 2:**
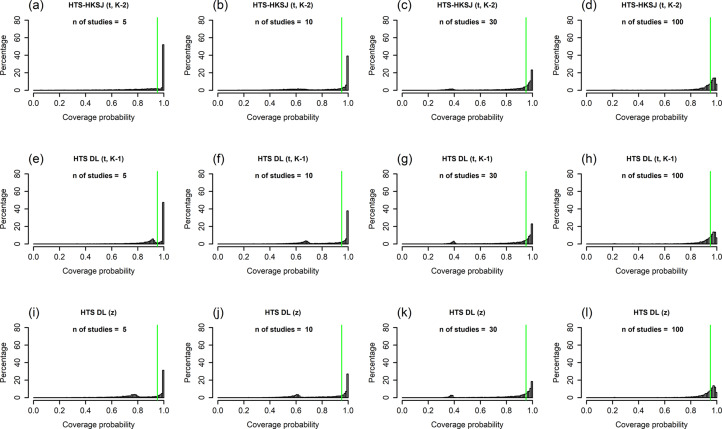
Histograms showing the coverage probability distribution of the HTS-HKSJ (t_K-2_), the HTS-DL (t_K-1_), and the HTS-DL (z) prediction interval methods for a low heterogeneity simulation scenario (N = 100, τ^2^ = 0.2, I^2^ = 33%, v = 0.5). The vertical green lines on the histograms indicate 95% coverage probability. The number of involved studies is constant in each column, showing the distribution for 5, 10, 30, and 100 studies. Results for the HTS-HKSJ (t_K-2_) method are represented in the first row of histograms with letters (a), (b), (c), and (d), the HTS-DL (t_K-1_) method is represented in the second row of histograms with letters (e), (f), (g), and (h), and the HTS-DL (z) method is represented in the third row of histograms with letters (i), (j), (k), and (l). *Abbreviations*: HTS-HKSJ (t_K-2_), Higgins–Thompson–Spiegelhalter method with the Hartung–Knapp–Sidik–Jonkman variance estimation for the μ parameter and using the t distribution with K–2 degrees of freedom; HTS-DL (t_K-1_), Higgins–Thompson–Spiegelhalter method with the DerSimonian and Laird estimation of 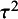 parameter and using the t distribution with K–1 degrees of freedom; HTS-DL (z), Higgins–Thompson–Spiegelhalter method with the DerSimonian and Laird estimation of 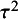 parameter and using the standard normal distribution.

**Figure 3:**
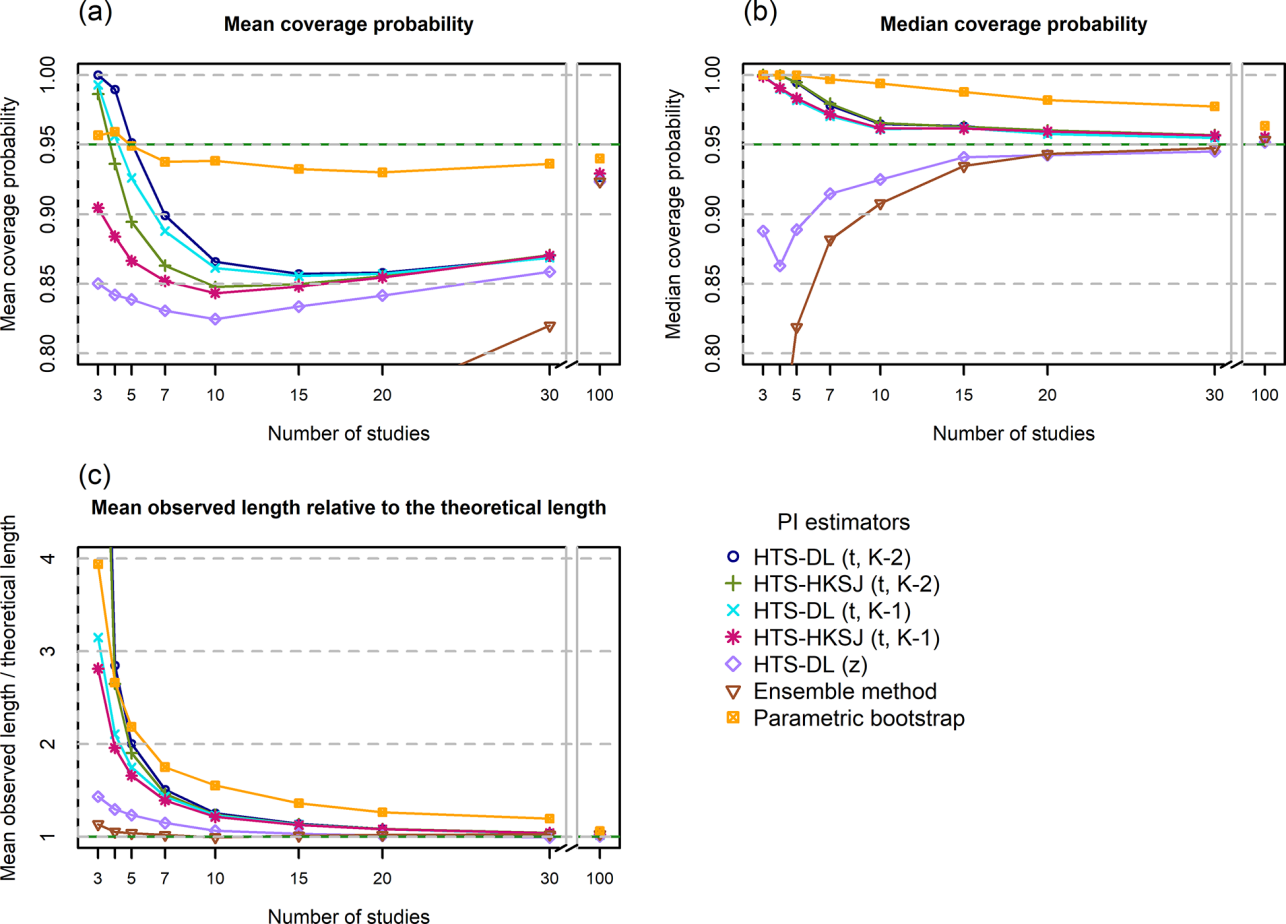
Mean coverage probability (a), Median coverage probability (b), and Mean observed length relative to the theoretical length (c) of the investigated prediction interval methods as a function of the number of involved studies (horizontal axis) for a low heterogeneity simulation scenario (N = 100, τ^2^ = 0.2, I^2^ = 33%, v = 0.5). *Abbreviations*: HTS, Higgins–Thompson–Spiegelhalter method; DL, DerSimonian and Laird estimation of 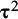 parameter; HKSJ, Hartung–Knapp–Sidik–Jonkman variance estimation for the μ parameter; t_K-2_, method calculated with t distribution with K-2 degrees of freedom; z, method calculated with standard normal distribution.

**Figure 4:**
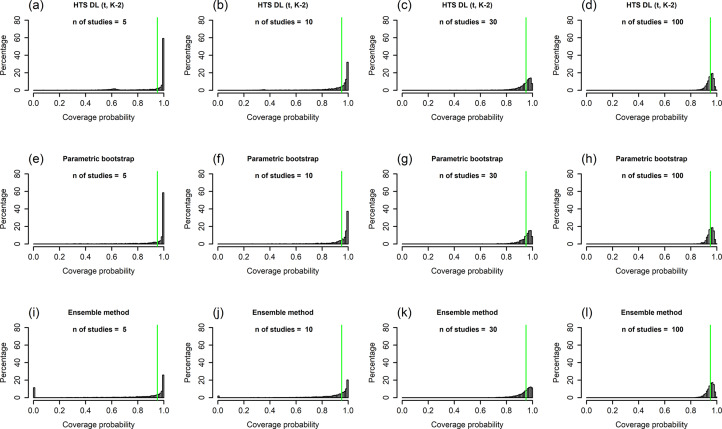
Histograms showing the coverage probability distribution of the HTS-DL (t_K-2_), the parametric bootstrap, and the ensemble prediction interval methods for a high heterogeneity simulation scenario (N = 100, τ^2^ = 1, I^2^ = 71%, v = 2.5). The vertical green lines on the histograms indicate 95% coverage probability. The number of involved studies is constant in each column, showing the distribution for 5, 10, 30, and 100 studies. Results for the HTS-DL (t_K-2_) method are represented in the first row of histograms with letters (a), (b), (c), and (d), the parametric bootstrap method is represented in the second row of histograms with letters (e), (f), (g), and (h), and the ensemble method is represented in the third row of histograms with letters (i), (j), (k), and (l). *Abbreviations*: HTS-DL (t_K-2_), Higgins–Thompson–Spiegelhalter method with the DerSimonian and Laird estimation of 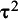 parameter and using the t distribution with K-2 degrees of freedom.

**Figure 5:**
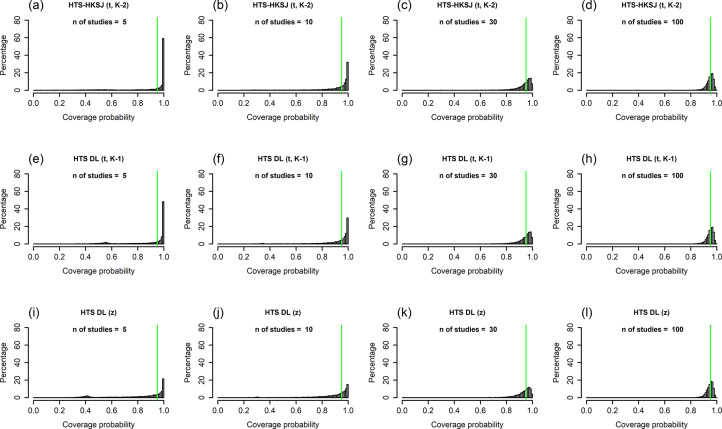
Histograms showing the coverage probability distribution of the HTS-HKSJ (t_K-2_), the HTS-DL (t_K-1_), and the HTS-DL (z) prediction interval methods for a high heterogeneity simulation scenario (N = 100, τ^2^ = 1, I^2^ = 71%, v = 2.5). The vertical green lines on the histograms indicate 95% coverage probability. The number of involved studies is constant in each column, showing the distribution for 5, 10, 30, and 100 studies. Results for the HTS-HKSJ (t_K-2_) method are represented in the first row of histograms with letters (a), (b), (c), and (d), the HTS-DL (t_K-1_) method is represented in the second row of histograms with letters (e), (f), (g), and (h), and the HTS-DL (z) method is represented in the third row of histograms with letters (i), (j), (k), and (l). *Abbreviations*: HTS-HKSJ (t_K-2_), Higgins–Thompson–Spiegelhalter method with the Hartung–Knapp–Sidik–Jonkman variance estimation for the μ parameter and using the t distribution with K-2 degrees of freedom; HTS-DL (t_K-1_), Higgins–Thompson–Spiegelhalter method with the DerSimonian and Laird estimation of 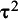 parameter and using the t distribution with K-1 degrees of freedom; HTS-DL (z), Higgins–Thompson–Spiegelhalter method with the DerSimonian and Laird estimation of 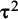 parameter and using the standard normal distribution.

**Figure 6:**
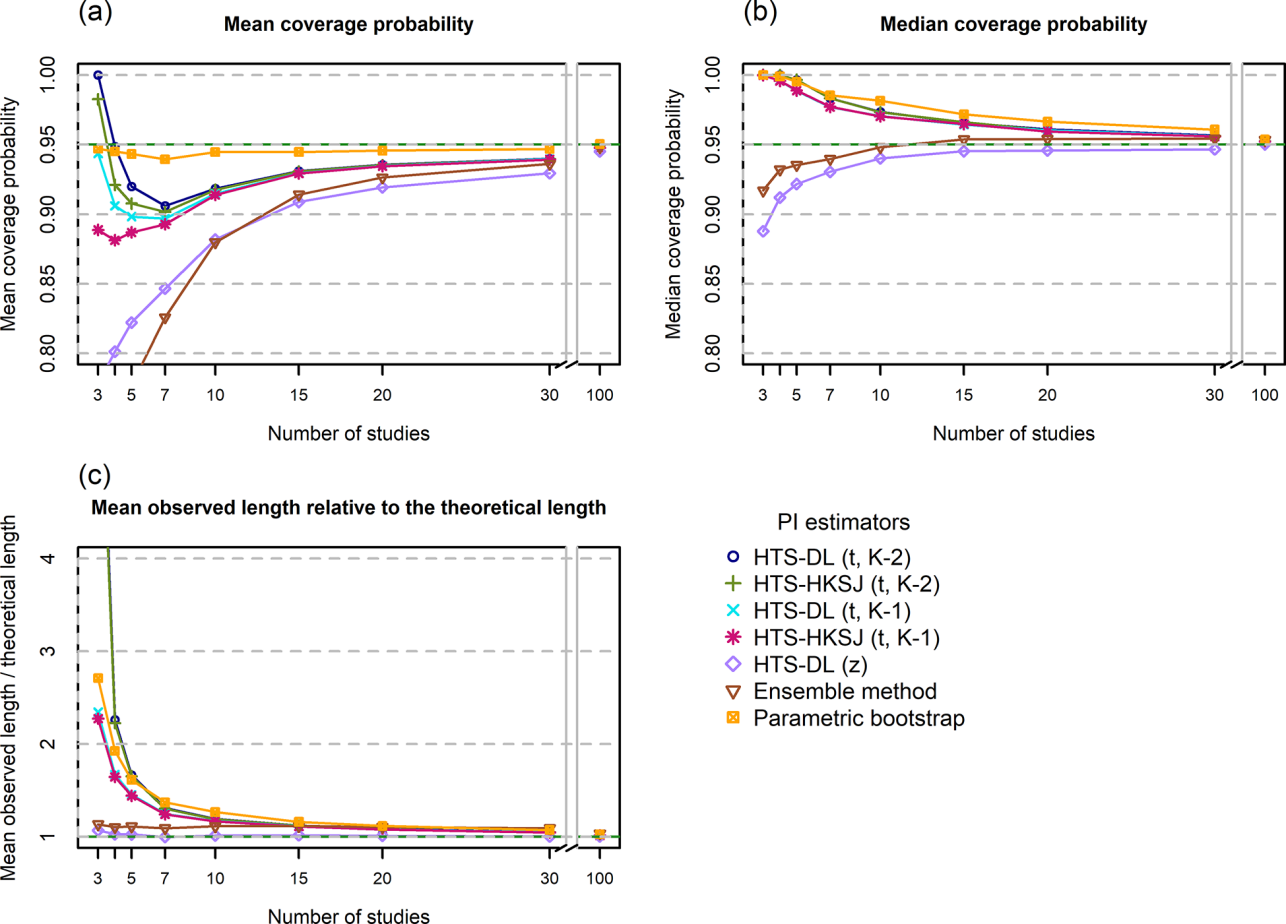
Mean coverage probability (a), Median coverage probability (b), and Mean observed length relative to the theoretical length (c) of the investigated prediction interval methods as a function of the number of involved studies (horizontal axis) for a high heterogeneity simulation scenario (N = 100, τ^2^ = 1, I^2^ = 71%, v = 2.5). *Abbreviations*: HTS, Higgins–Thompson–Spiegelhalter method; DL, DerSimonian and Laird estimation of 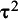 parameter; HKSJ, Hartung–Knapp–Sidik–Jonkman variance estimation for the μ parameter; t_K-2_, method calculated with t distribution with K-2 degrees of freedom; z, method calculated with standard normal distribution.

All of our simulation results are available in our Online Material file.^33^ This is a compact and easy-to-use HTML file, which contains all performance measure plots for all of our simulation scenarios. We included figures for two scenarios in the main text and the Supplementary Material: a low heterogeneity scenario (Figures 1– 3: N = 100, τ^2^ = 0.2, I^2^ = 33%, v = 0.5) and a high heterogeneity scenario (Figures 4– 6: N = 100, τ^2^ = 1, I^2^ = 71%, v = 2.5).

In Sections 5.1–5.4, we describe results for the coverage distribution, for the mean and median coverage, and the mean observed interval length relative to the theoretical length. In the Supplementary Material, we present figures with interpretation about two other performance measures (mean absolute difference from 95% coverage and normalized mean absolute error, Appendix Figures 3, 4 and Appendix Texts 1, 2 of the Supplementary Material). In Sections 5.1–5.4, for all the presented and discussed scenarios, the random effects are normally distributed. In Section 5.5, we discuss the impact of the non-normality of the random effects. The HTS-REML (t_K-2_) method gave almost identical results compared to the HTS-DL (t_K-2_) method; therefore, we present and describe results only for the latter method. Histograms for the HTS-HKSJ (t_K-1_) method can be found in the Appendix Figures 1 and 2 in the Supplementary Material.

### 5.1 Histogram of coverage probabilities

The histograms of coverage probabilities show the same pattern for each method: for any given sample size and τ^2^ combination, the histograms are very left-skewed unless the number of involved studies is extremely large (K = 100). As there are more and more studies in the meta-analysis, the coverage distributions get less and less left-skewed and start cumulating on the nominal level of 95%, showing an ideal, fairly symmetric shape. This pattern is true in general for all scenarios, but for smaller heterogeneity (Figures 1 and 2), this process is slower, and even for a large number of studies, the distributions remain left-skewed. For higher heterogeneity scenarios (Figures 4 and 5), as the number of studies increases, the coverage distributions reach a concentrated, less skewed shape faster. For low heterogeneity scenarios, it can be observed that the distributions of the HTS type PIs are multi-modal; for example, the HTS-DL (t_K-2_) method with K = 30 studies produces a mode of about 0.4 coverage (Figure 1c). The cause of this phenomenon is that the τ^2^ parameter estimate is often 0 in these cases, which frequently results in low coverage. The 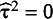 cases are also the reason why the ensemble method gives a mode coverage probability of 0 for scenarios with low heterogeneity and low study number (Figure 1i).

### 5.2 Mean coverage probability

The HTS (t) methods give a very high, nearly 100% mean coverage for K = 3 studies, and then as the number of studies increases, their mean coverage drops below the nominal level. For low heterogeneity, these methods are unable to produce the nominal mean coverage, and when the number of involved studies is extremely large (K = 100), the mean coverage of these methods is still about 2–3% below 95% (Figure 3a). When the heterogeneity is larger (Figure 6a), this drop is less dramatic, and they yield a mean coverage close to the nominal level even for K = 15 studies. The HTS-DL (z) and the ensemble methods give a very low mean coverage for the lower heterogeneity scenarios, and they only retain mean coverages close to the nominal level when both heterogeneity and the number of studies are large. The mean coverage of the parametric bootstrap method remains close to the nominal level irrespective of heterogeneity and study number. This method is able to produce mean coverages close to 95% even when the included number of studies is very low (K = 3–5).

### 5.3 Median coverage

The HTS (t) and the parametric bootstrap methods yield a higher than 95% median coverage in general, and they only retain 95% median coverage for scenarios with very large study numbers (Figures 3b and 6b). The parametric bootstrap method tends to give higher median coverages than the HTS (t) methods, especially for the lower heterogeneity scenarios. The HTS-DL (z) and the ensemble methods give a median coverage below 95% especially for the lower heterogeneity, lower study number scenarios, and as the study number increases, they approach the 95% level from below 95%.

### 5.4 Mean observed length relative to the theoretical length

The HTS (t) and the parametric bootstrap methods produce, on average, much longer intervals than the theoretical length, especially when the number of studies is low. As the study number increases, their mean observed length gets closer and closer to the theoretical length (Figures 3c and 6c). The parametric bootstrap method tends to give even longer intervals on average than the HTS (t) methods, especially when the heterogeneity is lower. The HTS-DL (z) and the ensemble methods yield a shorter mean observed interval compared to the HTS (t) and bootstrap methods when the number of involved studies is low (K < 15).

### 5.5 Impact of non-normality

When the true effects distributions depart from normal, the histograms are similar to the case when they follow the normal distribution. The only exception is where the random effects are drawn from the uniform distribution. In this case, a spike remains at >99% coverage for all methods irrespective of other circumstances, and the histograms do not approach a concentrated, symmetric form as the number of studies increases.^33^

The mean and median coverages are very similar to the normal distribution case described above when the true effects distribution is skewed or bimodal. For the uniform scenario, methods give higher mean coverage compared to the normal case, and the median coverage is nearly 100% regardless of any other parameters. Methods tend to give slightly higher mean observed length compared to the theoretical length when the true effects have a non-normal distribution, especially when their distribution is uniform.

## 6 Example

**Figure 7:**
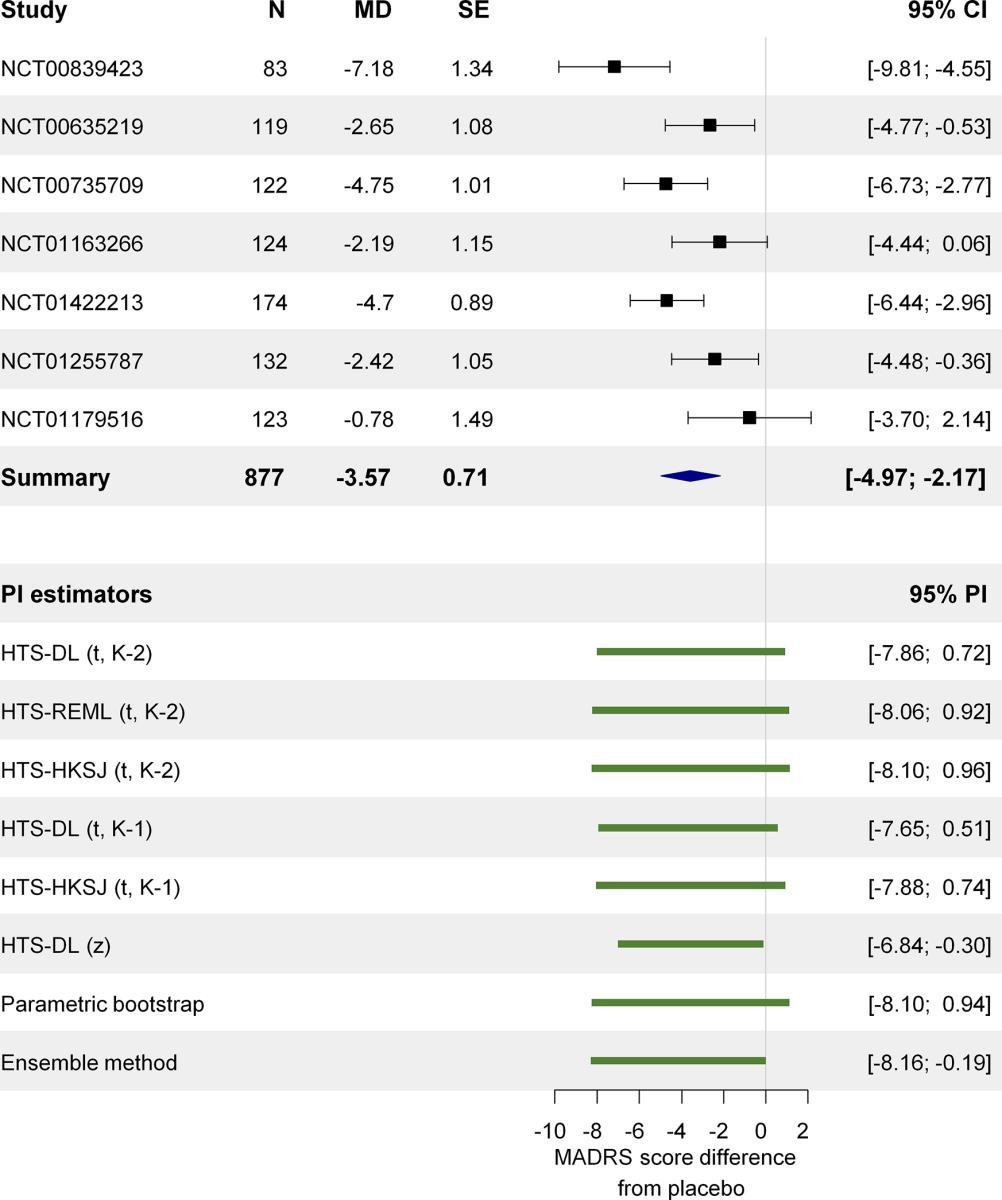
Forest plot showing the short-term efficacy of the active agent vortioxetine 10 mg in adult patients suffering from major depressive disorder based on the meta-analysis of Thase and his co-authors.^35^ *Abbreviations*: N, total sample size; MD, mean difference; SE, standard error; CI, confidence interval; PI, prediction interval; MADRS, Montgomery–Åsberg Depression Rating Scale; HTS, Higgins–Thompson–Spiegelhalter method; REML, Restricted maximum likelihood estimation of 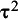 parameter; DL, DerSimonian, and Laird estimation of 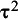 parameter; HKSJ, Hartung–Knapp–Sidik–Jonkman variance estimation for the μ parameter; t_K-2_, method calculated with t distribution with K-2 degrees of freedom; z, method calculated with standard normal distribution.

We chose a highly cited meta-analysis conducted in the medical field to illustrate the place of the prediction interval in the interpretation of the summary results and also the arising concerns. Thase and his co-authors published a meta-analysis in 2016 investigating the short-term (6–8 weeks) efficacy of the active agent vortioxetine in adult patients suffering from major depressive disorder.^35^ Their analysis included 11 randomized, double-blind, placebo-controlled trials investigating the effect of fixed doses of vortioxetine of 5, 10, 15, and 20 mg. Not each trial had study arms with each dose, so we chose their 10 mg analysis because it included the most trials, 7. The primary endpoint was the mean change from baseline relative to placebo in the MADRS total score. The MADRS is a depression rating scale ranging between 0 and 60, with higher points indicating more severe depression. A reduction of two points on the scale compared to placebo is generally considered clinically meaningful.^35^ Based on the study level data that they present in their Figure 2a, we reconstructed their 10 mg dose analysis. Thase et al. do not reveal it in their article, but based on their published numbers, we think they used the DL method to compute the summary statistics and the I^2^ measure. Based on the random-effects model, the mean effect with its 95% CI shows a clear advantage of vortioxetine over placebo: 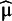 = −3.57, 95% CI: −4.97; −2.17. However, there is substantial heterogeneity: the I^2^ measure is 65%, the observed effects of the individual studies range between −7.18 and − 0.78. There is one study (NCT00839423) that has a CI with no overlapping part with the CIs of three other studies. In this case, reporting only the 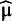 and its CI masks these important differences between the individual studies; however, reporting the PI reveals them. We computed the 95% PI with each method described in Section 3.2. (Figure 7). The PI computed with any method is much wider than the CI; the widest is the parametric bootstrap PI (−8.1; 0.94). The HTS (t) intervals are just slightly shorter, and they also cross the null effect line. Only the HTS-DL (z) and the ensemble methods give a PI that does not pass the null effect line. These PIs therefore suggest that in some populations, which are similar to the ones represented in these seven studies, a null effect or even a harmful effect of 10 mg vortioxetine is possible.

In the following part of this section, we examine our simulation results for a scenario very similar to this particular example (N = 100, I^2^ = 71%, K = 7 studies). The histograms show that the probability of a 95% PI with an extremely high level of coverage (>99%) is 20–50% depending on the chosen method (see the Online Material file^33^). The mean coverage is about 94% for the bootstrap method, and about 80–90% for the other methods. The median coverage of the bootstrap and the HTS (t) methods for this scenario is 98–99%, meaning that in 50% of the cases, these methods give a 95% PI that has a coverage of at least 98–99% (Figures 6a and 6b). Our length analysis reveals for this scenario that, on average, the bootstrap and the HTS (t) methods give 30% wider intervals than the theoretical length, the ensemble method gives about 10% wider intervals compared to the theoretical length, and the HTS-DL (z) method retains the theoretical length (Figure 6c).

This means that for this particular case, the only method that fulfills the PI criterion of retaining the average nominal coverage is the bootstrap method. In this sense, this is a reliable method because it fulfills its obligation in terms of mean coverage, while the other methods can be called unreliable, because these produce a mean coverage not more than 90% (Figure 6a). However, this does not mean that the parametric bootstrap gives a PI with near 95% coverage every time it is constructed; the very left-skewed histogram and the median coverage of 99% clearly reveal this (Figure 6b). It is important to understand that the coverage criterion has to be fulfilled only on average in repeated sampling, and the coverage probability of one single PI can seriously differ from this.

## 7 Discussion

Although the only condition that a prediction interval has to fulfill is to retain a nominal coverage probability on average, we argue that assessing only the mean coverage might lead to oversimplification and possible misunderstanding. The most complete and correct understanding of the behavior and interpretation of this interval can be achieved by also investigating the distribution of the coverage probabilities.

Our simulation results confirm the findings of Nagashima et al.^8^ regarding the parametric bootstrap method achieving the nominal level of mean coverage even if the number of involved studies is very small, for example, K = 3–5 (Figure 3a). The problem gets clear if we look at the histogram of coverages, for example, the K = 5 scenario of the parametric bootstrap method in Figure 4e. Here, we can see that in 60% of the cases, the PI computed by this method covers more than 99% of the true effects distribution. It gives a coverage close to 95% very rarely, but eventually it produces a mean coverage of 95%. It is able to yield a mean nominal coverage because the few cases of very low, 20–60% coverages balance out the overwhelming majority of coverages exceeding even 99%. Unfortunately, this pattern remains stable when the number of involved studies is larger (Figure 4f) and reaches a close to ideal form only for scenarios with a very large number of studies. In Figure 4h, which shows the distribution for high heterogeneity and K = 100 studies, we can see that the parametric bootstrap method gives 95% coverage not only on average, but we can expect that all the individual coverages will be close to 95% as well. This is the basic pattern and the general phenomenon with all PI methods, but we wanted to describe it with the parametric bootstrap method because this retains near nominal coverage on average, regardless of the amount of heterogeneity or study number. The other methods are frequently not able to maintain it, even on average.

The above-described findings have very serious implications for any researcher who is conducting a meta-analysis and wants to use the PI to summarize the results. The common interpretation of the PI is that this is an interval of “plausible” ^9^ or “expected” ^2^ ^,^ ^6^ range of true effects in similar studies or simply that this is a “range of true effects in future studies”.^12^ We think that researchers or readers of meta-analyses might misinterpret the above or similar definitions and believe that this is an interval that covers 95% of the true effects distribution. The distribution of coverages clearly shows that, unfortunately, this interpretation is only valid on average, and cannot be generalized for a certain case of meta-analysis, unless the number of involved studies is extremely large .

The very left-skewed distribution of PI coverages gives an explanation why so many meta-analyses with statistically significant mean parameters give a PI that contains the null effect or even the opposite value of the mean effect. IntHout et al. re-evaluated 479 statistically significant MAs from the Cochrane Database published between 2009 and 2013. They found that 72% of these have a 95% PI that contains the null effect, and 20% of the calculated PIs contain the opposite effect, suggesting that the effect in similar populations could be even the opposite, that is, harmful.^2^ Siemens et al. conducted a similar study analyzing MA studies in the 2010–2019 period, searching in multiple databases and identified 261 MAs with a significant mean effect. They report that in 75% of these, the 95% PI contains the null effect, and in 38% it contains the opposite effect.^3^ The explanation for this large proportion of PIs crossing the no effect line or even containing clinically relevant harmful effect values can be found in the low number of involved studies. Our analysis of median coverages shows that PIs tend to give more coverage than intended in the majority of MAs. We showed that this is especially true for MAs with a low number of studies. IntHout et al. re-analyzed studies with a median study number of K = 4,^36^ and the median study number in Siemens et al. is K = 6.^3^ In light of our analysis, their findings are not surprising but rather anticipated.

Some authors argue that the PI is helpful for the power calculation of a new trial.^2^ ^,^ ^6^ We think that whenever the PI is used in the planning of a new study, it is important to keep in mind that the coverage probability of that particular PI can seriously differ from the nominal level. This is a clear example where the analyst is interested in the coverage probability of that single PI they have at hand, and the mean coverage is unimportant. Therefore, we discourage the use of the PI for this purpose.

It is possible to perform a meta-analysis from a Bayesian perspective and compute a Bayesian PI. Bayesian MA approaches assume prior distributions for the mean and variance parameters of the true effects distribution, and compute the data-given posterior marginal of the mean parameter via MCMC simulation. Then, to get a PI in this framework, it is possible to extend the joint likelihood of the parameters and the observed data with the true effect of a new study. The 0.025 and 0.975 quantiles of this additional true effect’s posterior distribution form the 95% Bayesian PI. See Higgins et al.,^5^ Hamaguchi et al.,^10^ and Chapter 6.8 of Schmid et al.^37^ for more details. The interpretation of the frequentist and Bayesian PI is similar. The difference is that in the frequentist framework, the true effect of a new study is assumed to follow the same true effects distribution as the other involved studies, while in the Bayesian framework, it is assumed to follow the marginal posterior distribution. In particular, it is also true for Bayesian approaches that the coverage criterion must be fulfilled only on average. See Hamaguchi et al.^10^ for the frequentist performance of the Bayesian PI in terms of mean coverage and interval length.

Although it is rarely used in the context of the RE MA, it is possible to define an interval with more ambitious goals than the PI: the tolerance interval. With the notation used in Section 3, a random interval (L(D), U(D)) is a β-content tolerance interval if P [C ≥ β] = 1 – α, where β is the interval content (coverage proportion) and 1 – α is the confidence level. Brannick et al.^1^ argue convincingly that many times the tolerance interval suits to the given research question better because the prediction interval aims to contain a certain proportion of the true effects only *on average*; however, the tolerance interval aims to contain *at least this proportion* with some predefined confidence level.^38^

There are different thresholds proposed in the literature for the number of involved studies under which the use of PI is not advisable. Partlett and Riley conclude that if the heterogeneity is large enough (I^2^ > 30%) and the study sizes are balanced, the HTS type methods perform well if K ≥ 5.^9^ Nagashima et al. state that their bootstrap method is valid even if the number of included studies is very small and performs well even when K = 3.^8^ Siemens et al. claim that reporting the PI for K ≥ 4 helps decision-making.^3^ The current version of the Cochrane Handbook (version 6.5) encourages researchers to use the PI when K ≥ 5 and if there is no sign of funnel plot asymmetry.^7^

Based on our findings, we do not think it is advisable to calculate and present the PI for a meta-analysis with K < 15 studies. For an MA with smaller heterogeneity (I^2^ < 50%), we advise using the parametric bootstrap method if K ≥ 30. For higher heterogeneity MAs (I^2^ > 50%), we suggest presenting the PI if the MA contains at least 15 studies. In this case, we also propose using the parametric bootstrap method. Another aspect in the choice is considering which error type has milder consequences. We suggest using the parametric bootstrap method if predicting a larger interval than the theoretical one is less harmful. If it is more important to avoid predicting a too large interval, we suggest one of the HTS methods. We do not propose using the ensemble method in its current form because it is not able to produce an interval if 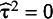 . Another valid consideration for choosing an HTS-type PI method can be to use the same τ^2^ estimator and distribution for the calculation of both the CI and the PI.^6^ ^,^ ^7^ This approach has the advantage that in the absence of observed heterogeneity, the CI and the PI are identical.

We encourage practitioners to inspect the PI performance measures in our Online Material file^33^ for those parameters they assume in their own analysis. By doing so, they can assess the properties of the PI methods for their own case.

We agree with Brannick et al. that without sufficient information, we cannot anticipate accurate estimates and that in these cases it is better not to report any PI.^1^ Cox^39^ and Higgins^5^ argue that for a small number of studies, it is worth considering reporting only the individual study results and not reporting any summary statistics. Our analysis clearly shows that this is particularly true for the PI.

## 8 Conclusions

Our aim with this study was to conduct an in-depth analysis of the performance of the PI in the frequentist random-effects meta-analysis and to help researchers understand its correct interpretation.

We think that researchers and readers of meta-analyses might misinterpret the PI in a way that it has a near nominal coverage every time it is constructed. Inspecting the coverage distributions reveals that this interpretation is only valid if the number of involved studies is extremely large. In case of an MA with small study numbers, we cannot expect that one particular computed PI will have near nominal coverage, even if we know that it fulfills the criterion of keeping the nominal mean coverage. Further research is needed on how an interval can be constructed or altered to fulfill more strict conditions than the simple mean coverage criterion.

## Supporting information

10.1017/rsm.2025.10055.sm001 Mátrai et al. supplementary material Mátrai et al. supplementary material

## Data Availability

All R codes used for the simulation are available on GitHub: https://github.com/peter-matrai/Sim_Matrai.git.

## Supplementary material

To view supplementary material for this article, please visit http://doi.org/10.1017/rsm.2025.10055.
