# Supplementary material for: Assessing the properties of the prediction interval in random-effects meta-analysis
Source: Res Synth Methods. 2026 Jan 9;17(3):517–37. doi: 10.1017/rsm.2025.10055 (PMC13126221; doi:10.1017/rsm.2025.10055)
Supplement: Mátrai et al. supplementary material [file S1759287925100550sup001.pdf]

Appendix for the article

Assessing the properties of the prediction interval in random-effects meta-analysis

Péter Mátrai[1,2], Tamás Kói[3,4], Zoltán Sipos[1,2], Nelli Farkas[1,2]

[1] Institute of Bioanalysis, Medical School, University of Pécs, Pécs, Hungary

[2] Institute for Translational Medicine, Medical School, University of Pécs, Pécs, Hungary

[3] Department of Stochastics, Institute of Mathematics, Budapest University of Technology and Economics, Budapest, Hungary

[4] Centre for Translational Medicine, Semmelweis University, Budapest, Hungary

APPENDIX PROOF 1

Proof that the expected value of the covered probability C, defined in section 3.1. in equation (3) fulfills equation (4)

In this section we will prove that the expected value of the covered probability C, defined in (3) fulfills (4). For the sake of completeness, first we review important properties about the conditional expectation based on Chapter 4.1. in Durrett's textbook Probability: Theory and Examples [1].

When Y is a random variable on the probability space (Ω; $\mathcal{F}_0$; P) with $E(|Y|) < \infty$ and $\mathcal{F}$ is a sub sigma-algebra of $\mathcal{F}_0$ then the conditional expectation $E(Y|\mathcal{F})$ is almost surely well defined. In the sequel all equalities between random variables mean almost sure equality. In particular, when $\mathcal{F}$ is the trivial sigma-algebra, i.e., $\mathcal{F} = \{\emptyset, \Omega\}$ then $E(Y|\mathcal{F})$ is equal to the conventional expected value E(Y).

If both X and Y are random variables on the probability space (Ω; $\mathcal{F}_0$; P), then the conditional random variable E(Y|X) can be reformulated as $E(Y|X)=E(Y|\sigma(X))$, where $\sigma(X)$ is the sigma-algebra generated by X, i.e., the smallest sigma-algebra according to which X is measurable. It will be crucial in the upcoming proof that the probability of an event $A \in \mathcal{F}_0$ can be expressed as

$$P(A) = E(\mathbb{1}_A), \tag{7}$$

where $\mathbb{1}_A$ is the indicator function of event A, i.e., the random variable on (Ω; $\mathcal{F}_0$; P) defined by

$$1_A(\omega) = \begin{cases} 1 & \omega \in A \\ 0 & \omega \notin A \end{cases} . \tag{8}$$

Note also that P(A|X) can be defined as

$$P(A|X) = E(\mathbb{1}_A|X). \tag{9}$$

Proposition 1: (Example 4.1.7. in Durrett [1]) Suppose X and Y are independent. Let ϕ be a function with $E(|\phi(X, Y)|) < \infty$ and let $g(x) = E(\phi(x, Y))$. Then

$$E(\phi(X, Y)|X) = g(X). \quad (10)$$

Proposition 2: (Special case of formula (4.1.5) in Durrett [1], also known as tower rule). Let X and Y be random variables on the probability space $(\Omega; \mathcal{F}_0; P)$ with $E(|Y|) < \infty$. Then

$$E(E(Y|X)) = E(Y). \quad (11)$$

To prove (4), first observe that the independence of Θ_{New} and data D, equation (9) and Proposition 1 imply that

$$P[\Theta_{New} \in (L(D), U(D))|D] = E[1_{\Theta_{New} \in (L(D), U(D))}| D] = E[\phi(\Theta_{New}, D)|D] =$$

$$F(U(D)) - F(L(D)) = C. \quad (12)$$

Finally, the above equality, (8), (9) and Proposition 2 imply

$$E[C] = E\left[E\left[1_{\Theta_{New} \in (L(D), U(D))}\middle|D\right]\right] = \quad (13)$$

$$E[1_{\Theta_{New} \in (L(D), U(D))}] = \quad (14)$$

$$P[\Theta_{New} \in (L(D), U(D))]. \quad (15)$$

Hence, the claimed equality (4) is proved.

Reference:

[1] Durrett, R. (2019). Probability: Theory and Examples (5th ed.). Cambridge: Cambridge University Press.

APPENDIX FIGURE 1

Histograms showing the coverage probability distribution of the HTS-HKSJ (t_{K-1}) prediction interval method for a **low heterogeneity** simulation scenario (N=100, τ^2=0.2, I^2=33%, v=0.5). The vertical green lines on the histograms indicate 95% coverage probability. The number of involved studies are 5, 10, 30 and 100, represented by letters (a), (b), (c) and (d), respectively.

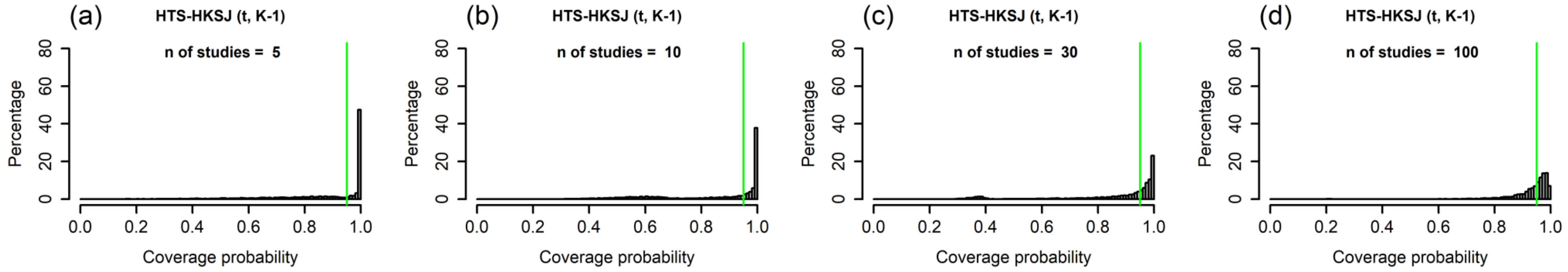

Abbreviations: HTS-HKSJ (t_{K-1}), Higgins – Thompson – Spiegelhalter method with the Hartung - Knapp - Sidik – Jonkman variance estimation for the μ parameter and using the t distribution with K-1 degrees of freedom.

APPENDIX FIGURE 2

Histograms showing the coverage probability distribution of the HTS-HKSJ (t_{K-1}) prediction interval method for a **high heterogeneity** simulation scenario (N=100, τ^2=1, I^2=71%, v=2.5). The vertical green lines on the histograms indicate 95% coverage probability. The number of involved studies are 5, 10, 30 and 100, represented by letters (a), (b), (c) and (d), respectively.

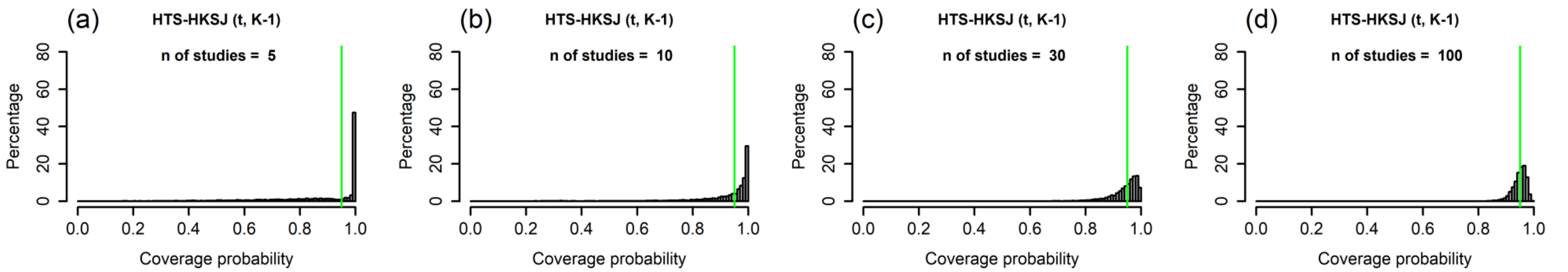

Abbreviations: HTS-HKSJ (t_{K-1}), Higgins – Thompson – Spiegelhalter method with the Hartung - Knapp - Sidik – Jonkman variance estimation for the μ parameter and using the t distribution with K-1 degrees of freedom

APPENDIX FIGURE 3

Mean absolute difference from 0.95 coverage (a) and Normalized mean absolute error (b) of the investigated prediction interval methods as a function of the number of involved studies (horizontal axis) for a **low heterogeneity** simulation scenario (N=100, τ^2=0.2, I^2=33%, v=0.5).

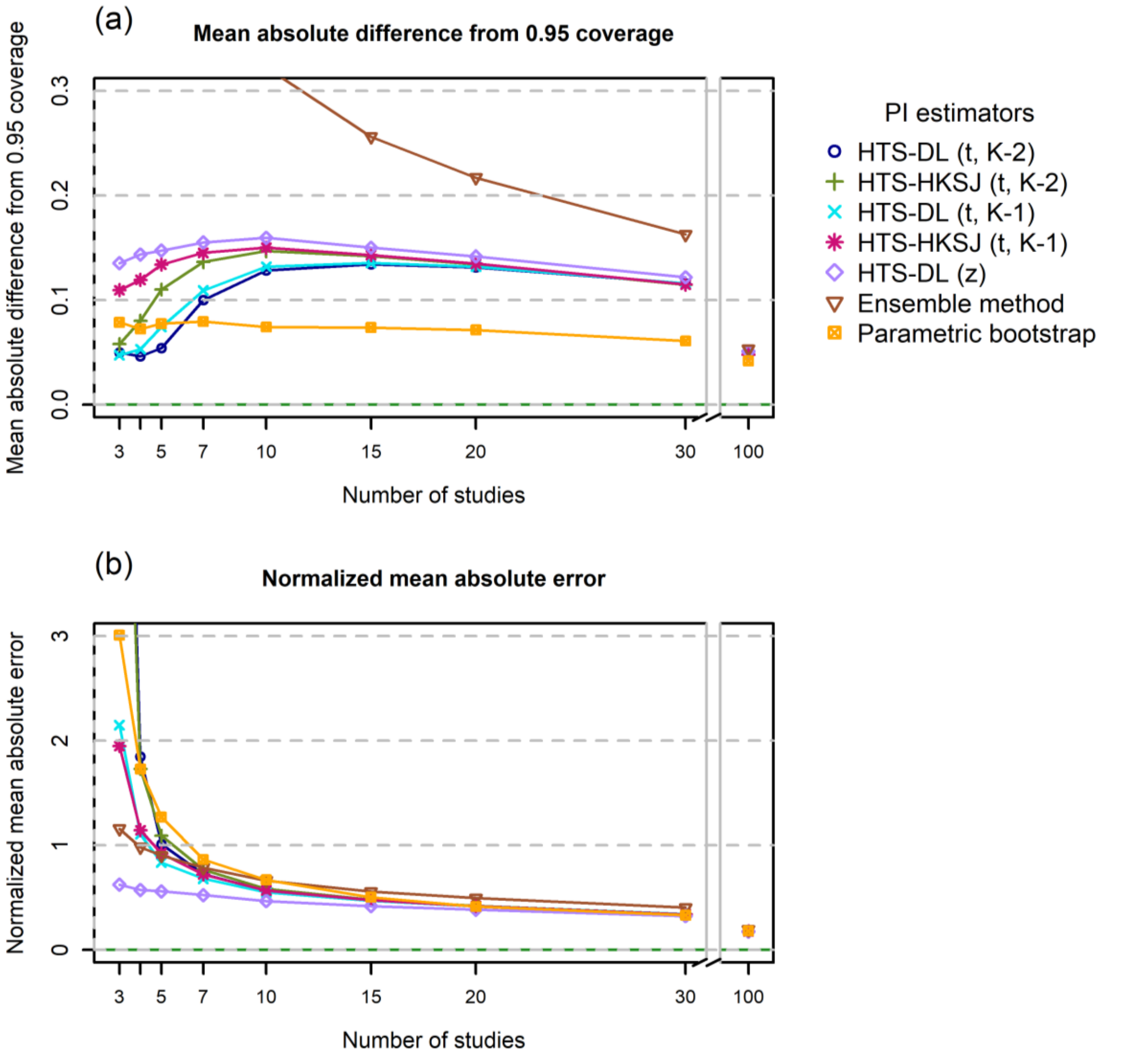

Abbreviations: HTS, Higgins – Thompson – Spiegelhalter method; DL, DerSimonian and Laird estimation of τ^2 parameter; HKSJ, Hartung - Knapp - Sidik – Jonkman variance estimation for the μ parameter; t_{K-2}, method calculated with t distribution with K-2 degrees of freedom; z, method calculated with standard normal distribution

APPENDIX FIGURE 4

Mean absolute difference from 0.95 coverage (a) and Normalized mean absolute error (b) of the investigated prediction interval methods as a function of the number of involved studies (horizontal axis) for a **high heterogeneity** simulation scenario (N=100, τ^2=1, I^2=71%, v=2.5).

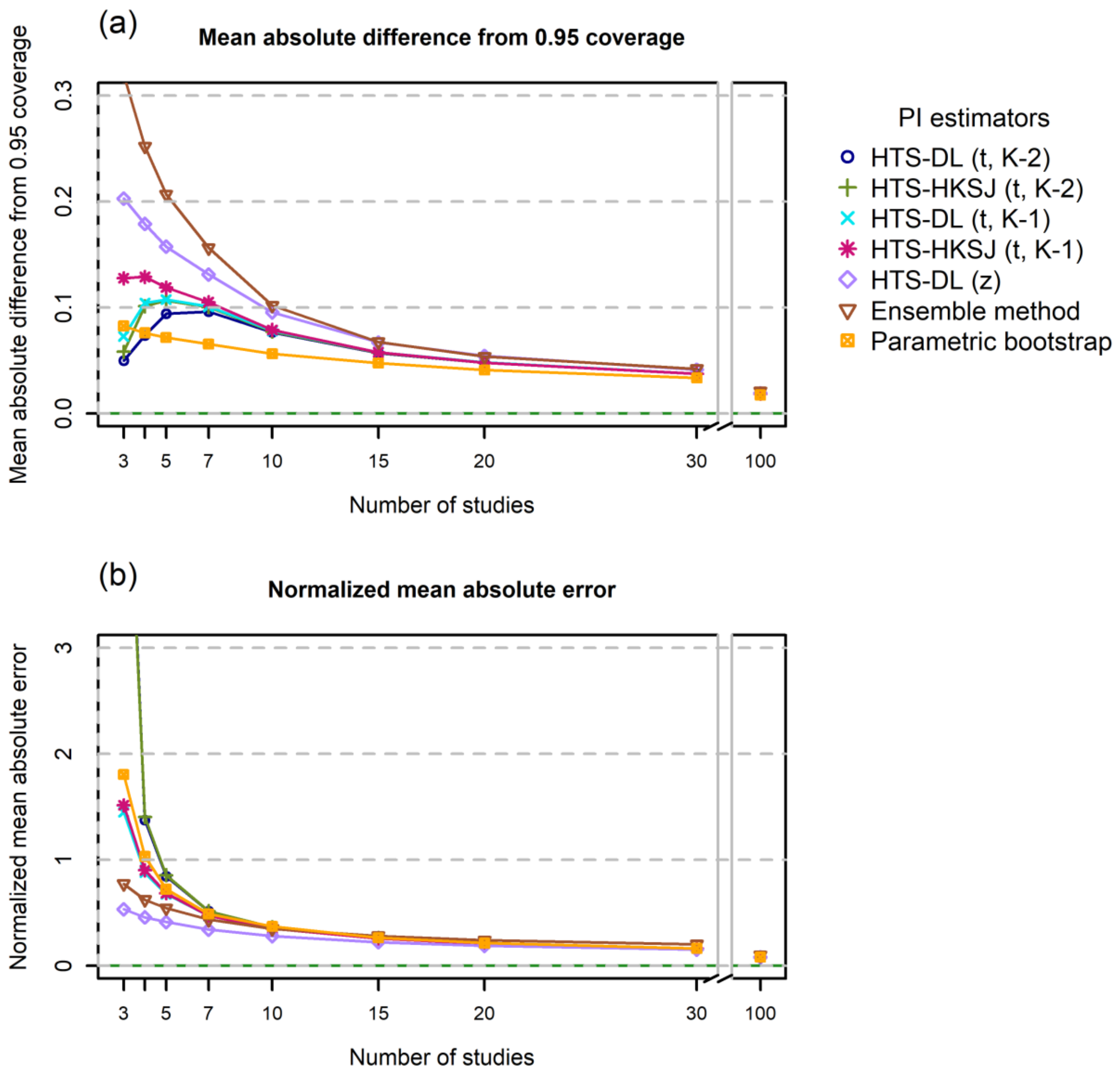

Abbreviations: HTS, Higgins – Thompson – Spiegelhalter method; DL, DerSimonian and Laird estimation of τ^2 parameter; HKSJ, Hartung - Knapp - Sidik – Jonkman variance estimation for the µ parameter; t_{K-2}, method calculated with t distribution with K-2 degrees of freedom; z, method calculated with standard normal distribution

APPENDIX TEXT 1

Mean absolute difference from 95% coverage

The mean absolute difference from 95% coverage approximates the quantity $E[\,|C - 0.95|\,]$. The HTS (t) methods give about a 5% mean absolute difference for the K=3 study number scenarios (Appendix Figures 3a and 4a). For the higher study number scenarios this increases, especially for the lower heterogeneity cases (Appendix Figure 3a), and for scenarios with very large study number (K=100) it gets closer to 0 again, but even for K=30, they give about a 10% mean absolute difference from the nominal coverage. For higher heterogeneity scenarios (Appendix Figure 4a), this initial increase is smaller and the mean absolute difference remains smaller than it is for the small heterogeneity cases. The HTS-DL (z) method gives a higher mean absolute difference than the HTS (t) intervals for the lower study number scenarios, just as the ensemble method. For higher heterogeneity and study number scenarios this difference gets smaller and when $v \geq 5$ and $K \geq 15$ each method yields very similar mean absolute difference. The parametric bootstrap method gives the smallest mean absolute difference of all methods, except the very low study number scenarios (K = 3-5).

APPENDIX TEXT 2

Normalized mean absolute error

The normalized mean absolute error, or phrased differently the mean relative distance from the theoretical length approximates the quantity $\mathrm{E}\left[\,\left|\frac{\ell - \mathrm{T}}{\mathrm{T}}\right|\,\right]$. The parametric bootstrap and the HTS (t) methods give a relatively high mean absolute error if the study number is low (K < 7) and if the study number increases, this error decreases (Appendix Figures 3b and 4b). The HTS-DL (z) and the ensemble methods give smaller mean absolute error for these low study number scenarios compared to the other investigated methods. If the heterogeneity is high, (Appendix Figure 4b) all methods produce smaller normalized mean absolute error compared to the low heterogeneity scenarios (Appendix Figure 3b).
